# Supplementary material for: 3-D phononic crystals with ultra-wide band gaps
Source: Sci Rep. 2017 Feb 24;7:43407. doi: 10.1038/srep43407 (PMC5324071; doi:10.1038/srep43407)
Supplement: Supplementary Information [file srep43407-s1.pdf]

Supplementary information

File format

The mesh and material distribution data of optimization results are written in the form of the open source software *ParaView* files with suffix *.vtk* and contained in *.zip* files. These supplementary data can be opened and edited as normal text files in *Notepad* or *gedit*. Each *.vtk* file includes 4 parts of data. For a given optimized model with n elements along each primitive cell vector, the 1st part of the data contains $(n+1)^3$ nodal coordinates, the 2nd part provides the number of nodes within each element and the nodal connectivity, the 3rd part is the element type indicator, where the number 12 stands for block element, and the final part is the material distribution, where 0 means tungsten carbide and 1 means epoxy in this study.

Visualization

To visualize the data, open the *.vtk* files in *ParaView* as shown in Supplementary Fig. 1. To separate the two materials, click the ”Threshold” function in the ”Common” toolbar and change the threshold values under the ”Properties” tab.

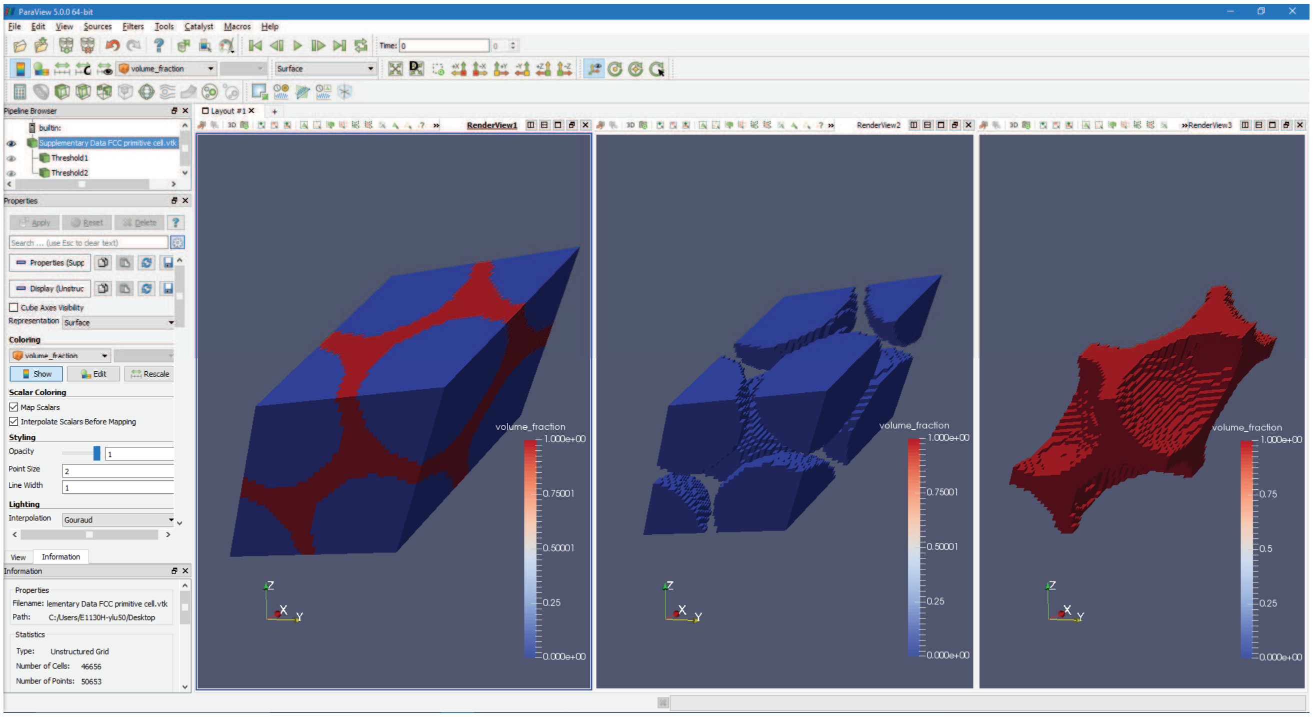

Supplementary Figure 1. *ParaView* interface with optimized FCC primitive cell file opened.
